# Synthesis of borocarbonitride nanosheets from biomass for enhanced charge separation and hydrogen production

**DOI:** 10.1038/s41598-024-65380-y

**Published:** 2024-06-23

**Authors:** Zhishan Luo, Jinhao Chen, Yuanmeng Fang, Liyan Xie, Qing Liu, Jianhui Huang, Minghua Liu

**Affiliations:** 1 Fujian Provincial Key Laboratory of Ecology-Toxicological Effects and Control for Emerging Contaminants, College of Environmental and Biological Engineering, Putian University, Putian, 351100 China; 2 Key Laboratory of Ecological Environment and Information Atlas, Fujian Provincial University (Putian University), Putian, 351100 China; 3 State Key Laboratory of Photocatalysis on Energy and Environment, College of Chemistry, Fuzhou University, Fuzhou, 350002 China; 4 College of Environment and Safety Engineering, Fuzhou University, Fuzhou, 350116 China

**Keywords:** Photocatalysis, Water splitting, Hydrogen production, Borocarbonitride (BCN), Biomass synthesis, Catalyst synthesis, Photocatalysis

## Abstract

Borocarbonitride (BCN) materials have shown significant potential as photocatalysts for hydrogen production. However, traditional bulk BCN exhibits only moderate photocatalytic activity. In this study, we introduce an environmentally conscious and sustainable strategy utilizing biomass-derived carbon sources to synthesize BCN nanosheets. The hydrogen evolution efficiency of BCN-A nanosheets (110 μmol h^−1^ g^−1^) exceeds that of bulk BCN photocatalysts (12 μmol h^−1^ g^−1^) by 9.1 times, mainly due to the increased surface area (205 m^2^g^−1^) and the presence of numerous active sites with enhanced charge separation capabilities. Notably, the biomass-derived BCN nanosheets offer key advantages such as sustainability, cost-effectiveness, and reduced carbon footprint during hydrogen production. These findings highlight the potential of biomass-based BCN nanomaterials to facilitate a greener and more efficient route to hydrogen energy, contributing to the global transition towards renewable energy solutions.

## Introduction

In response to the escalating costs and deepening crisis of fossil fuels, there is an urgent need to explore alternative, renewable energy sources^1^. Solar energy stands out for its cleanliness, safety, and inexhaustibility, qualities that are widely recognized and valued^2–4^. The pioneering work of Fujishima and Honda, who demonstrated the photoelectrochemical splitting of water into H_2_ and O_2_ using a TiO_2_ anode and a Pt cathode under ultraviolet irradiation^5^, has led to extensive studies on the photocatalytic reactions of light-emitting semiconductor particles aimed at converting solar energy into chemical energy^6–9^. One particularly promising approach is the conversion of solar energy into hydrogen through water splitting, facilitated by semiconductor-based photocatalysis^10,11^. However, designing an efficient photocatalyst that is cost-effective, abundantly available, environmentally sustainable, and capable of maintaining high charge carrier separation mobility for efficient photocatalysis remains a significant challenge.

Recent years have seen the intentional design of conjugated polymers^12^, including covalent organic frameworks (COFs)^13–17^, conjugated triazine frameworks (CTFs)^18–22^, polymeric carbon nitrides (PCN)^23–30^, conjugated microporous polymers (CMPs)^31–34^, and BCN-based polymers^35–40^, as promising metal-free photocatalysts due to their semiconductive, electrochemical, and optical properties. Among these, BCN has emerged as a highly promising candidate, distinguished by its unique electronic and structural properties that facilitate both CO_2_ fixation and water-splitting reactions^41^. Significant progress has been made through modulating BCN's dimensions and textures, encompassing applications such as nanotubes^42^, nanosheets^43^, 3D structures^44^, and composite materials. Despite these advancements, most BCN materials do not achieve optimal photocatalytic performance due to limitations in surface area, optical absorption, crystallinity, and charge recombination, with bulk BCN photocatalysts lacking structural and morphological optimization resulting in minimal activity. Thus, the future of photocatalytic development hinges on the careful design and control of BCN's structural, morphological, and electronic properties.

The synthesis of BCN photocatalysts can be achieved through various methods. Conventional approaches often face challenges related to costly and scarce carbon sources. For instance, the synthesis of BCN semiconductors frequently employs the chemical vapor deposition (CVD) technique, necessitating the use of reagents like boron trichloride (BCl_3_), ammonia (NH_3_), and acetylene (C_2_H_2_)^45^. Plasma-assisted CVD, on the other hand, utilizes source gases such as BCl_3_ and methane (CH_4_) for BCN synthesis^46^. These methodologies are inherently limited in their widespread applicability due to the substantial costs and scarcity of these essential reactants.

To address this issue, our research introduces a biomass-directed synthesis strategy for BCN nanosheets, leveraging abundant and renewable carbon sources derived from biomass. This approach offers an appealing and environmentally friendly alternative to traditional BCN synthesis methods. Biomass, recognized as the most abundant renewable carbon-rich resource^47^, serves as a significant reservoir of carbon precursors suitable for BCN fabrication^48,49^. While numerous studies have reported on the use of biomass for BCN photocatalyst synthesis, most have utilized only one type of biomass carbon, such as glucose^50–52^. There is a dearth of research exploring the use of multiple types of biomass carbon to synthesize a range of BCN photocatalysts, with even fewer systematic investigations into the variations in composition, structure, and performance among them. To bridge this gap, our study selects diverse types of biomass as carbon sources for BCN photocatalyst synthesis and subsequently compares the differences in their morphology, composition, structure, and performance. This methodology not only addresses concerns about the scarcity and cost associated with conventional carbon sources but also plays a crucial role in the carbon cycle and contributes to advancing knowledge in synthesizing BCN photocatalysts from diverse biomasses as carbon sources and understanding their distinctions. The exploration of biomass-derived BCN nanosheets as photocatalysts may set the stage for scalable and eco-friendly hydrogen production technologies, promoting carbon recycling and reducing greenhouse gas emissions, aligning with the principles of sustainable development and contributing to a cleaner, more resilient energy future.

## Results and disscussion

### Synthesis and characterizaiton of BCN photocatalysts

**Figure 1:**
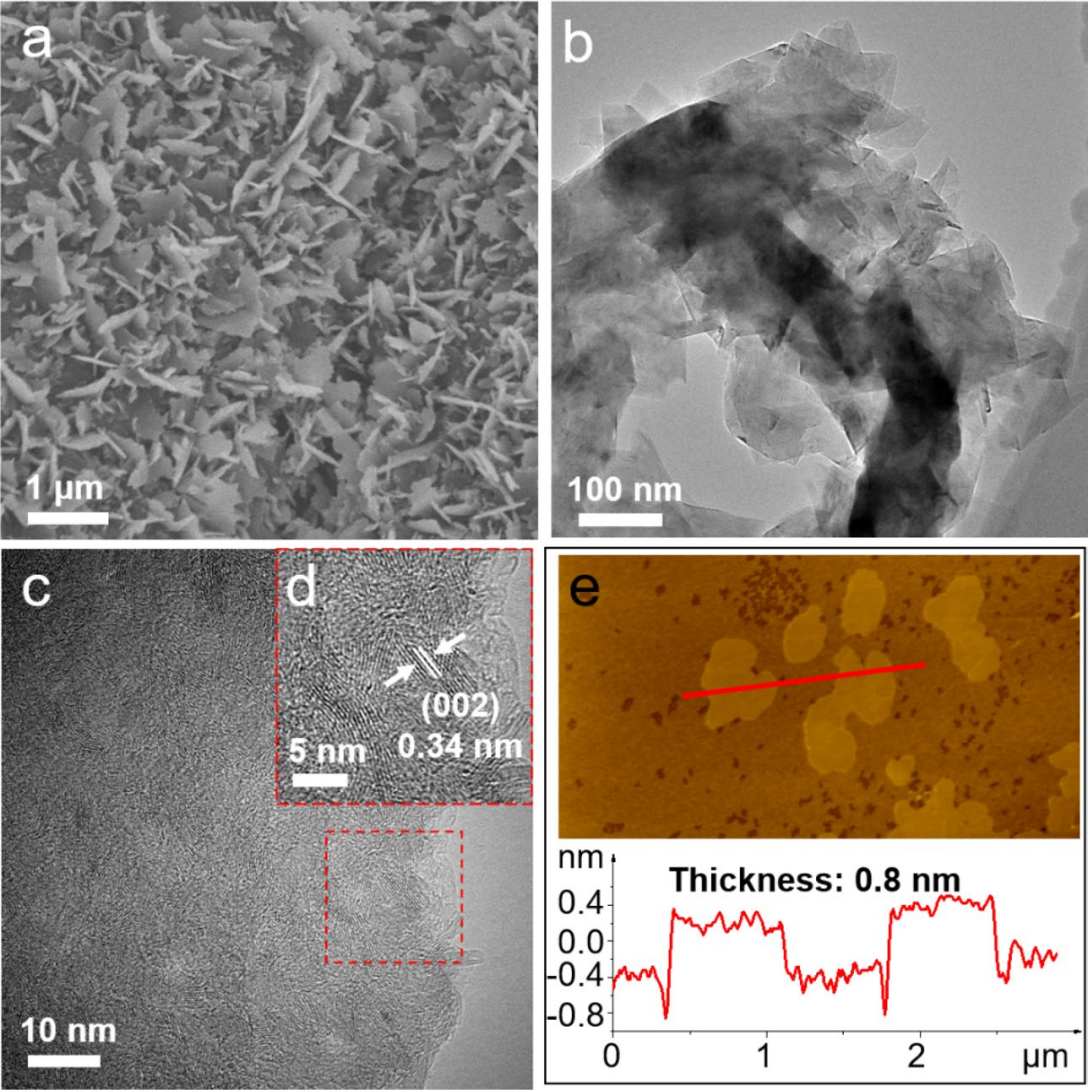
The morphology of BCN photocatalysts. (**a**) SEM, (**b**, **c**) TEM, (**d**) High-resolution TEM and (**e**) AFM images of BCN-A catalysts.

Borocarbonitride (BCN) photocatalysts were synthesized through a high-temperature ammoniation method. Biomass materials, namely amylum, sucrose, maltose, and fructose, were employed as carbon sources, resulting in the designation of BCN-A, BCN-S, BCN-M, and BCN-F photocatalysts, respectively. Detailed information regarding the synthesis of these BCN photocatalysts is provided in the "Method" section. The morphology of these BCN photocatalysts was analyzed using scanning electron microscopy (SEM), transmission electron microscopy (TEM), and atomic force microscopy (AFM). The investigation revealed that BCN photocatalysts, synthesized employing amylum, exhibit a two-dimensional (2D) sheet structure. As illustrated in Fig. 1a, BCN-A photocatalysts manifest a nanosheet morphology. In contrast, BCN-S, BCN-M, and BCN-F exhibit bulk morphologies, as depicted in Supplementary Fig. 1a,d, and g, respectively. The low-magnification TEM images in Fig. 1b, Supplementary Fig. 1b,e, and h further confirm the nanosheet structure of BCN-A photocatalysts, while the others exhibit a bulk-like configuration. Figures 1c and d present a remarkable high-resolution transmission electron microscopy (HR-TEM) image of the BCN-A photocatalysts. Notably, the (002) facet of BCN-A displays distinct, well-ordered graphite-like layers with a precise lattice spacing of 0.34 nm^53^. In contrast, the HR-TEM contrast intensity profiles of the other BCN photocatalysts (Supplementary Fig. 1c,f,i) do not provide discernible lattice fringes. This observation suggests a significantly improved crystallinity and reduced defect density in BCN-A photocatalysts. Furthermore, the thickness of BCN-A photocatalysts is determined to be approximately 0.8 nm using the Atomic Force Microscopy (AFM) technique (Fig. 1e), revealing a characteristic flake structure that aligns with the SEM findings. Subsequently, Energy-dispersive X-ray spectroscopy (EDS) experiments are conducted, as illustrated in Supplementary Fig. 2, reveal that all BCN photocatalysts exhibit a homogeneous distribution of boron (B), carbon (C), and nitrogen (N) elements. The uniform distribution observed underscores the consistent structural integrity of the BCN photocatalysts. The chemical formulae of these photocatalysts, predicated on the elemental analyses derived from EDS data, are delineated in Supplementary Table 1. It is crucial to emphasize that the presence of oxygen (O), whether in trace amounts or as a dopant, does not disqualify the material. This is especially pertinent considering the nuanced and variable aspects of synthesis processes, which can inadvertently result in unintended doping. As a result, the ultimate composition may deviate from the nominal B:C:N ratio of 1:1:1^54^.

**Figure 2:**
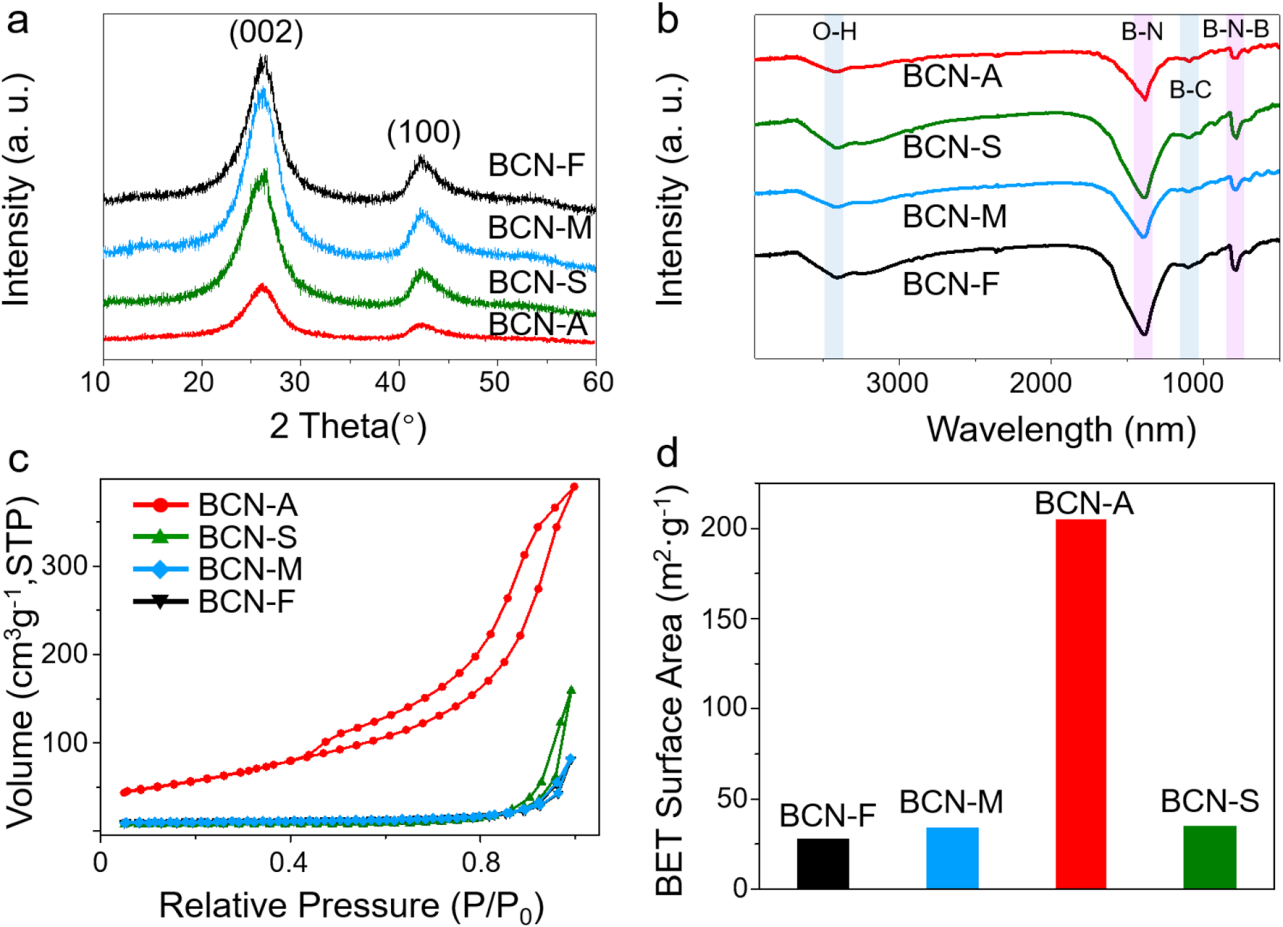
The XRD, FT-IR and BET spectra of BCN photocatalysts. (**a**) XRD patterns, (**b**) FT-IR spectra, (**c**) Nitrogen adsorption–desorption isotherms, and (**d**) surface area of BCN-A, BCN-S, BCN-M and BCN-F photocatalysts, respectively.

Subsequently, X-ray diffraction (XRD) patterns and Fourier-transform infrared (FT-IR) spectroscopy were employed for the characterization of these BCN photocatalysts. Figure 2a shows that all samples have the graphitic structure, as indicated by the presence of two distinct diffraction peaks at approximately 26.3° and 42.3°, corresponding to the (002) and (100) crystal planes, respectively^50^. The dominant peak at 26.3° represents the interlayer stacking characteristic of the graphite-like structure and correlates with a lattice fringe of 0.34 nm, as verified by the HR-TEM image shown in Fig. 1d ^44^. In particular, the decreased intensity of the (002) peak in the BCN-A photocatalyst clearly demonstrates the formation of layered BCN crystals^55^, which is consistent with the SEM and AFM results. Subsequently, the chemical framework of these BCN photocatalysts was examined using FT-IR spectra. As depicted in Fig. 2b, two characteristic infrared bands, positioned at 1390.8 and 790.3 cm^−1^, are associated with the in-plane transverse stretching vibrations of *sp*^2^-bonded B–N and the out-of-plane bending vibrations of B–N–B bands, respectively^56^. A broad absorption band observed at 3400 cm^−1^ across all BCN photocatalysts is ascribed to physically adsorbed H_2_O molecules. Notably, a minor absorption peak corresponding to B–C vibrations emerges at approximately 1091 cm^−1^, suggesting the integration of carbon atoms into the BN matrix. The FT-IR spectra results affirm the realization of atomic-level BCN hybrid structures^57^. Additionally, nitrogen adsorption–desorption isotherms and specific surface data for all BCN photocatalysts are presented in Fig. 2c,d and Supplementary Table 2, respectively. The specific surface area of BCN-A photocatalyst is the largest (205 m^2^g^−1^) compared to the other photocatalysts, suggesting the provision of more catalytically active sites for heterogeneous photoredox catalysis.

To further understand the structure of the BCN photocatalysts, we performed Raman spectroscopy. As shown in Supplementary Fig. 3, all the samples showed a wide D band around 1316 cm^−1^, highlighting the presence of disordered carbon in BCN. The intensity ratio (I_D_/I_G_) of the D band to the G band decreased from 0.85 (BCN-F) to 0.78 (BCN-A). This indicates that the BCN-A photocatalyst has relatively fewer defects and more complete and ordered crystal structure^54^, which is consistent with the high crystallinity shown by HR-TEM results.

To elucidate the chemical structure of BCN-A photocatalysts, X-ray photoelectron spectroscopy (XPS) experiments were conducted, as shown in Supplementary Fig. 4. The B 1s peaks can be deconvoluted into three peaks with binding energies centered at 190.2, 189.8, and 192.1 eV, corresponding to the B-N, B-C, and B-O bonds, respectively (Supplementary Fig. 4a)^57^. In the dominant C 1*s* spectrum in Supplementary Fig. 4b, a typical signal at 284.6 eV is attributed to graphitic carbon (C–C), while the shoulder peak at 285.8 eV indicates C–N bonds, and the weak signal at 283.8 eV is assigned to C–B bonds. The corresponding N-signals, depicted in Supplementary Fig. 4c, exhibit two distinguishable peaks centered at 397.8 and 398.7 eV, apportioned to the binding energies of N–B and N–C bonds, respectively.

### Optical and electrochemical properties

**Figure 3:**
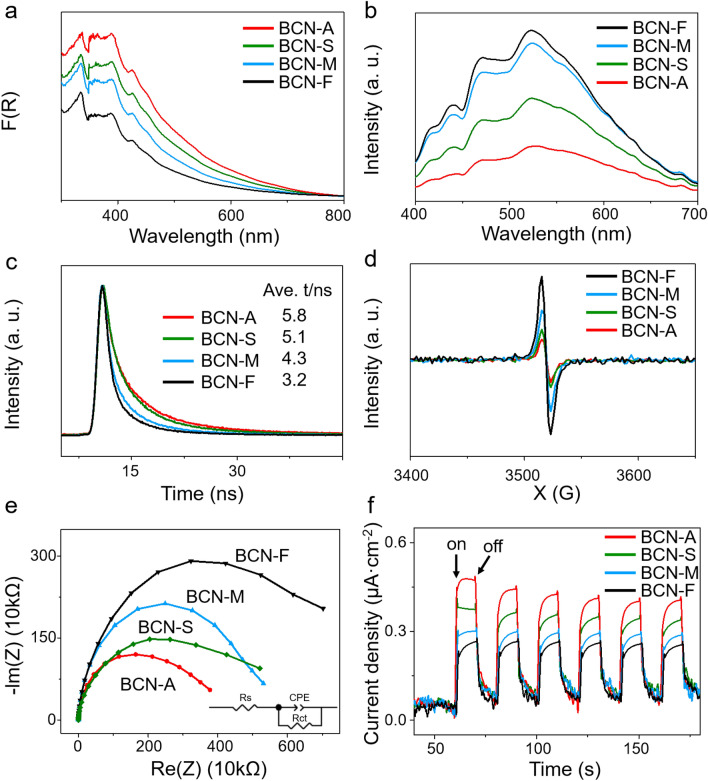
The photoelectrochemical properties. Images of (**a**) DRS, (**b**) PL, (**c**) PL lifetime decay, (**d**) EPR, (**e**) EIS and (**f**) TPR for BCN-A, BCN-S, BCN-M, and BCN-F photocatalysts, respectively.

To investigate the optical, electronic, and electrochemical characteristics of BCN photocatalysts, we conducted UV–vis diffuse reflectance spectroscopy (DRS), room temperature photoluminescence (PL), and electron paramagnetic resonance (EPR) spectra analyses. Notably, all BCN photocatalysts exhibit a discernible absorption range spanning from 300 to 800 nm (Fig. 3a). Remarkably, the absorption performance of the BCN-A photocatalyst surpasses that of the other BCN photocatalysts, suggesting that the nanosheet structure contributes to enhanced light absorption. Furthermore, the Kubelka–Munk function of BCN photocatalysts is illustrated in Supplementary Fig. 5, and the light absorption edge of BCN-A photocatalysts displays a noticeable redshift with the smallest band gap (2.77 eV), indicating a favorable photoresponsive behavior in BCN-A photocatalysts. The photoluminescence (PL) experiments, depicted in Fig. 3b, revealed a major emission wavelength of BCN photocatalysts at 523 nm upon excitation at 360 nm. Notably, in comparison with the other BCN photocatalysts, the PL intensity of BCN-A photocatalysts is significantly reduced, indicating that the 2D crystal structure promotes the separation and transfer of photogenerated carriers. The observed PL quenching in BCN photocatalysts is attributed to the low density of surface defects, known to typically act as recombination centers for separated electron–hole pairs^52^. Furthermore, as illustrated in Fig. 3c, the BCN-A photocatalyst exhibits a longer average PL lifetime (5.8 ns), revealing more efficient suppression of photoexcited charge recombination in foliated structures with low surface defects.

To further validate these findings, Electron paramagnetic resonance (EPR) measurements were conducted. As depicted in Fig. 3d, all BCN photocatalysts exhibit doublet EPR spectra located on the Lorentzian line with a g value equivalent to 2.0034, indicating the presence of unpaired electrons in the conduction band containing *sp*^2^-carbon orbitals^58^. The BCN-A photocatalyst exhibits a notably weaker EPR signal compared to other BCN photocatalysts. This observation aligns with the HR-TEM characterizations and suggests a reduced density of surface defects. Subsequently, experiments involving electrochemical impedance spectra (EIS) and transient photocurrent response were conducted to assess the charge transfer of BCN photocatalysts in both nanosheet and bulk-like structures. As depicted in Fig. 3e, an EIS experiment was conducted under dark conditions to scrutinize the surface charge transfer resistance of the BCN photocatalysts. An equivalent circuit, amalgamating solution resistance (Rs), charge transfer resistance (Rct), and a constant phase element (CPE), was devised for the purpose of dissecting the impedance spectra of the synthesized samples, as portrayed in the inset of Fig. 3e. The EIS spectrum of each sample was subsequently fitted and interpreted using Z-view software. In accordance with the fitting outcomes, the Rct magnitudes for the BCN-F, BCN-M, BCN-S, and BCN-A samples were ascertained to be 0.68, 0.49, 0.41, and 0.30 MΩ, respectively. Notably, the BCN-A photocatalyst manifests a comparatively diminished resistance relative to its counterparts. This observation substantiates the notion that the nanosheet architecture of the BCN photocatalysts potentially enhances electronic conductivity, thereby promoting efficient electron conveyance. In contrast, the photocurrent response outcomes for BCN photocatalysts are illustrated in Fig. 3f, showcasing the highest transient photocurrent density observed for the BCN-A photocatalyst upon illumination. This result indicates the more efficient separation of photo-generated electron–hole pairs on the surface of BCN nanosheets. Through the analysis above, the nanosheet structure exhibits characteristics such as high crystallinity, a large specific surface area, enhanced light absorption, low surface defects, and excellent charge separation and transfer. These attributes are anticipated to expedite photocatalytic reactions.

### Photocatalytic performance of BCN photocatalysts

**Figure 4:**
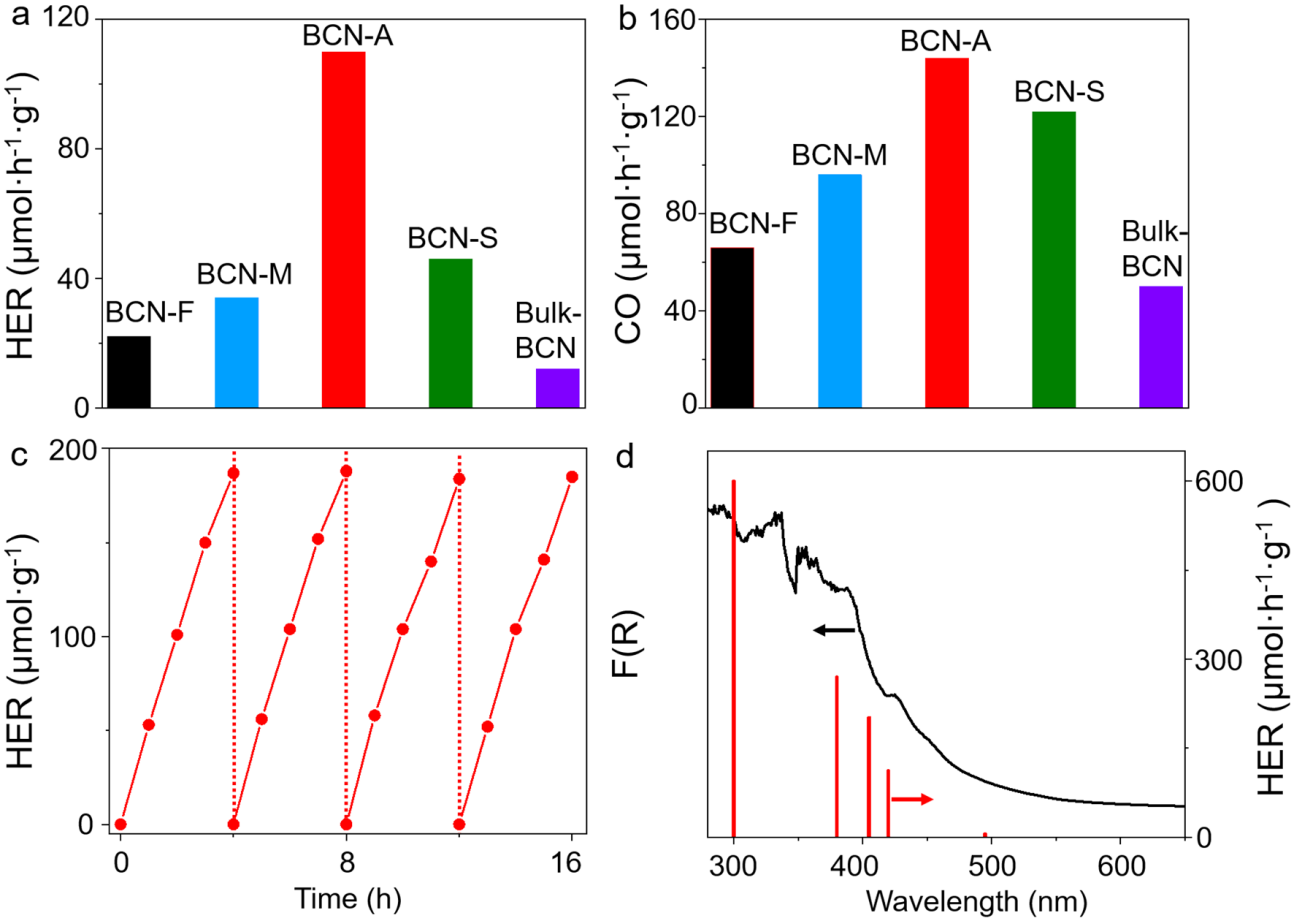
The hydrogen production and CO_2_ reduction performance. (**a**) The activity of hydrogen evolution reaction (λ > 420 nm), and (**b**) photocatalytic CO_2_ reduction performance (λ > 420 nm) for BCN photocatalysts. (**b**) A long time H_2_ generation test for BCN-A photocatalyst, and (**d**) wavelength-dependent photocatalytic H_2_ evolution rates for BCN-A photocatalyst under UV–vis and visible irradiation.

The evaluation of these BCN photocatalysts in photocatalytic hydrogen production under visible light irradiation was conducted, utilizing Pt nanoparticles as co-catalysts and triethanolamine (TEOA) as sacrificial reagents. The detailed descriptions of the photocatalytic process experiments are provided in the "Method" section. As depicted in Fig. 4a, the photocatalytic hydrogen evolution rate (HER) of BCN-A photocatalysts (110 μmol h^−1^ g^−1^) surpasses that of other BCN photocatalysts (Supplementary Table 3), representing at least a ninefold enhancement compared to bulk BCN photocatalysts (12 μmol h^−1^ g^−1^, the bulk BCN is synthesized without the incorporation of biomass, such as by using graphite as the carbon source, which exhibits compositional and structural attributes comparable to those of BCN-A photocatalysts, Supplementary Fig. 6), and was higher than that reported in the literature of other BCN photocatalysts (Supplementary Table 3). Moreover, we also test the photocatalytic CO_2_ reduction for BCN photocatalysts, and the BCN-A photocatalyst exhibits an enhancement performance when compared to the other BCN photocatalysts (Fig. 4b). This significant improvement can be attributed to the unique structural and electronic properties of the BCN-A nanosheets, including their increased specific surface area, which provides abundant active sites for photocatalytic reactions. These sites facilitate more efficient adsorption of reactant molecules and enhance the interaction between the catalyst and the reactants, thereby improving the overall photocatalytic efficiency. Additionally, the reduced impedance and increased photocurrent response of the BCN-A nanosheets, compared to other BCN photocatalysts, signify improved charge carrier dynamics. Further, the photocatalytic stability of BCN-A photocatalysts was assessed over 16 h with intermittent degassing every 4 h during the reaction. Figure 4c illustrates no noticeable deactivation under continuous illumination, suggesting the high stability of BCN-A nanosheets. This finding is further supported by structural examinations through XRD, FT-IR TEM, and XPS characterizations, revealing no significant structural changes in BCN-A photocatalysts before and after the extended stability test (Supplementary Fig. 7, 8 and 9)^51^. Furthermore, thermogravimetric analysis (TGA) showed that these BCN photocatalysts exhibited commendable thermal stability (Supplementary Fig. 10). Additionally, the wavelength-dependent photocatalytic H_2_ evolution rate of the BCN-A photocatalyst aligns well with its optical absorption behavior (Fig. 4d), indicating that the H_2_ evolution reaction is indeed driven by the light excitation of BCN-A nanosheets. Lastly, we have demonstrated that BCN nanosheets are successfully synthesized with the improved hydrogen production and CO_2_ reduction performance compared to other BCN photocatalysts.

## Conclusion

In summary, we select different types of biomass as carbon sources for BCN photocatalyst synthesis and subsequently compare the differences in their morphology, composition, structure, and performance. The resulting BCN-A nanosheet exhibits a significantly higher HER (110 μmol h^−1^ g^−1^) when compared to other BCN photocatalysts, which is mainly attributed to the synergistic effects of its unique electrical properties, flexible 2D structure, high crystallinity, and large specific surface area, facilitating the efficient separation and transfer of photoexcited electron–hole pairs. This study paved the way for the rational design and preparation of BCN nanosheets using various biomass sources as precursors within artificial photosynthesis systems. While limitations must be noted. Scalability poses a challenge, as current lab-scale methods may struggle with reaction control, product uniformity, and economic feasibility at industrial levels. Real-world applications also need to be tested, with factors like light exposure, temperature, and impurities affecting nanosheet stability and efficiency. Finally, the recyclability and environmental impacts of large-scale production must be studied. Addressing these limitations demands interdisciplinary collaborations, innovative techniques, and rigorous field testing to transition lab successes to large-scale applications.

## Methods

### Preparation of borocarbonitride

In a representative experiment, 5 g of amylum, 2 g of urea, and 1 g of boric acid were meticulously ground into fine powders. Subsequently, these precursors were introduced into a horizontal tube furnace. At this juncture, an aqueous solution containing 5 g of NaCl was added to the mixture before commencing the reaction. The assembled components were then subjected to heating at a temperature of 1250 °C, with a controlled heating rate of 5 °C min^−1^ and kept warm for 5 h under ammonia atmosphere by a flow rate of 200 mL min^−1^. The ammonia atmosphere was characterized by a concentration ranging from 99.6 to 99.8%, with the ammonia being stored in a cylinder of 40 L capacity. The cylinder was filled with 20 kg of ammonia and maintained an outlet pressure of 1 MPa. After cooling down, the collected product was grinded to powder and washed thoroughly with water and ethanol three times to remove any residual impurities. The obtained sample was denoted as BCN-A samples. The synthesis of BCN-S, BCN-M, BCN-F, and bulk BCN samples were similar to that described above, except that the amylum was replaced by sucrose, maltose, fructose, and graphite, respectively.

### Photocatalytic test for hydrogen evolution system

The photocatalytic hydrogen production test was carried out in a Pyrex top-irradiation reaction vessel connected to a glass closed gas circulation system. In a typical procedure, 50 mg catalyst powders were dispersed in a 100 mL aqueous solution containing triethanolamine (10 vol.%) as sacrificial electron donor. Then, Pt (1.0 wt%) nanoparticles as co-catalysts were loaded on the surface of catalysts by the in situ photo-deposition reaction of H_2_PtCl_6_ solution. The system was evacuated to remove air completely before irradiation with a 300 W Xenon lamp (λ > 420 nm, working current: 15 A, irradiation power density: 1.43 W/cm^2^). The temperature of the photocatalytic reaction process was controlled by circulating condensate at 12 °C. After 1 h of reaction, the generated gases were collected and analysed in situ by gas chromatography (GC: Shimadzu GC-8A; equipped with TCD detector; 5Å molecular sieve column; Argon as the carrier gas).

### Photocatalytic test for CO_2_ reduction system

The photocatalytic test was performed in a Schlenk flask (80 mL) under an atmospheric pressure of CO_2_. In the Schlenk flask, the photocatalytic CO_2_ reduction reaction was carried out by dispersing 50 mg catalyst in a solution containing solvent of H_2_O (2 mL) and acetonitrile (4 mL), triethanolamine (TEOA, 1 mL), CoCl_2_ (1 μmol) and 2,2-bipyridine (20 mg). This mixture system was subjected to vacuum degassing and then back filling with pure CO_2_ gas. This process was repeated three times, and after the last cycle, the flask was back filled with CO_2_ (1 bar). The temperature of the reaction solution was maintained at 30 °C controlled by a flow of warming water during the reaction. Then, the system was irradiated with a 300 W xenon lamp with a 420 nm cut-off filter under vigorous stirring. The produced gases (CO and H_2_) were detected using a gas chromatography equipped with a packed molecular sieve column (TDX-1 mesh 42/10). Argon was used as the carrier gas.

### Structure and composition characterization

Powder X-ray diffraction (XRD) patterns were detected with Bruker D8 Advance diffractometer with Cu-K1 radiation (λ = 1.5406 Å). The Fourier transform infrared (FT-IR) spectra were measured by a BioRad FTS 6000 spectrometer. Nitrogen adsorption–desorption isotherms were measured at 77 K using Micromeritics ASAP 3035 equipment. The pore size distribution was obtained by the Barrett-Joyner-Halenda (BJH) method and the specific surface area (SSA) was calculated from isotherms by the Brunauer–Emmett–Teller (BET) equation. The scanning electron microscope (SEM) characterizations were performed on a Hitachi SU8010 Field Emission Scanning Electron Microscope. The measurements of Transmission electron microscopy (TEM), including high-resolution electron microscopy (HR-TEM), high-angle-annular dark field (HAADF) images, and energy dispersive spectrometry (EDS) maps were carried out by a Thermo Fisher Scientific TEM (Talos F200S). The thermogravimetric analysis (TGA) was performed on SDT 650 (Waters Corporation). Raman spectroscopic measurements were performed on a Renishaw in Via Raman System 1000 with a 312 nm Nd:YAG excitation source at room temperature. Atomic force microscopy (AFM) measurements were performed in air using a Bruker ICON Atomic Force Microscope (Bruker Dimension Icon). The UV/Vis reflectance spectra (DRS) were measured by a Hitachi UH 5300 spectrophotometer. Photoluminescence (PL) spectra and time-resolved photoluminescence (TRPL) spectra were recorded using a Horiba Fluorolog-3 spectrophotometer. Electron paramagnetic resonance (EPR) experiments were conducted with a Bruker EPR A300 spectrometer. X-ray photoelectron spectroscopic (XPS) measurements were conducted wih an ESCALAB 250 (Thermo Fisher Scientific, USA) by using a monochromatized Al Kα line source (200 W). Z-View software (full name: ZView 4, version 4.0h, URL: https://www.scribner.com/software/68-general-electrochemistr376-zview-for-windows/).

### Photoelectrochemical measurements

Electrochemical experiments were conducted with a Biologic VSP-300 Electrochemical System in a typical three electrode cell setup with an electrolyte solution 0.2 M Na_2_SO_4_, using a Pt plate (counter electrode), and an Ag/AgCl electrode (reference electrode). The working electrode was prepared on fluorine-doped SnO_2_ (FTO) glass that was cleaned by sonication in water, acetone, and ethanol for 30 min and dried at 353 K. The FTO glass was fixed with scotch tapes, leaving a blank area of 0.25 cm^2^ to load BCN photocatalysts. The 5 mg BCN photocatalysts were dispersed in 1 mL of *N*,*N*-dimethylformamide (DMF) by ultrasound to give an ink mixture. The sample suspensions were dip-coated onto 0.25 cm^2^ pieces of FTO glass. After air-drying at room temperature, the scotch tape was removed, and the working electrode was further dried at 393 K for 2 h to improve adhesion. Finally, the uncoated part of the electrode was isolated with epoxy resin. Chronoamperometry (CA) curve was conducted by recording the photocurrent with a light 300 W xenon lamp (λ > 300 nm), and Electronic impedance spectra (EIS) were measured in a 0.2 M Na_2_SO_4_ aqueous solution without any irradiation, to obtain the intrinsic features of the flat-band potentials.

### Supplementary Information

Supplementary Information.

## Data Availability

The authors declare that the data supporting the findings of this study are available within the paper, and its supplementary information files.
