# Supplementary material for: Synthesis of borocarbonitride nanosheets from biomass for enhanced charge separation and hydrogen production
Source: Sci Rep. 2024 Jun 23;14:14443. doi: 10.1038/s41598-024-65380-y (PMC11194275; doi:10.1038/s41598-024-65380-y)
Supplement: Supplementary file 1 — Supplementary Information. [file 41598_2024_65380_MOESM1_ESM.docx]

**Supplementary Information for**

**Synthesis of borocarbonitride nanosheets from biomass for enhanced charge separation and hydrogen production**

Zhishan Luo^1-3^*, Jinhao Chen^1,2^, Yuanmeng Fang^1,2 4^, Liyan Xie^1,2^, Qing Liu^1,2^, Jianhui Huang^1,2^*and Minghua Liu^1,2,4^

^1^Fujian Provincial Key Laboratory of Ecology-Toxicological Effects & Control for Emerging Contaminants, College of Environmental and Biological Engineering, Putian University, Putian 351100, China.

^2^Key Laboratory of Ecological Environment and Information Atlas, Fujian Provincial University (Putian University), Putian 351100, China.

^3^State Key Laboratory of Photocatalysis on Energy and Environment, College of Chemistry, Fuzhou University, Fuzhou 350002 (China).

^4^College of Environment & Safety Engineering, Fuzhou University, Fuzhou 350116, China.

*To whom correspondence should be addressed.

Email: [zsluo@ptu.edu.cn](mailto:yuzyemlab@fzu.edu.cn); owenhuang95@163.com;

**Supplementary Figures**


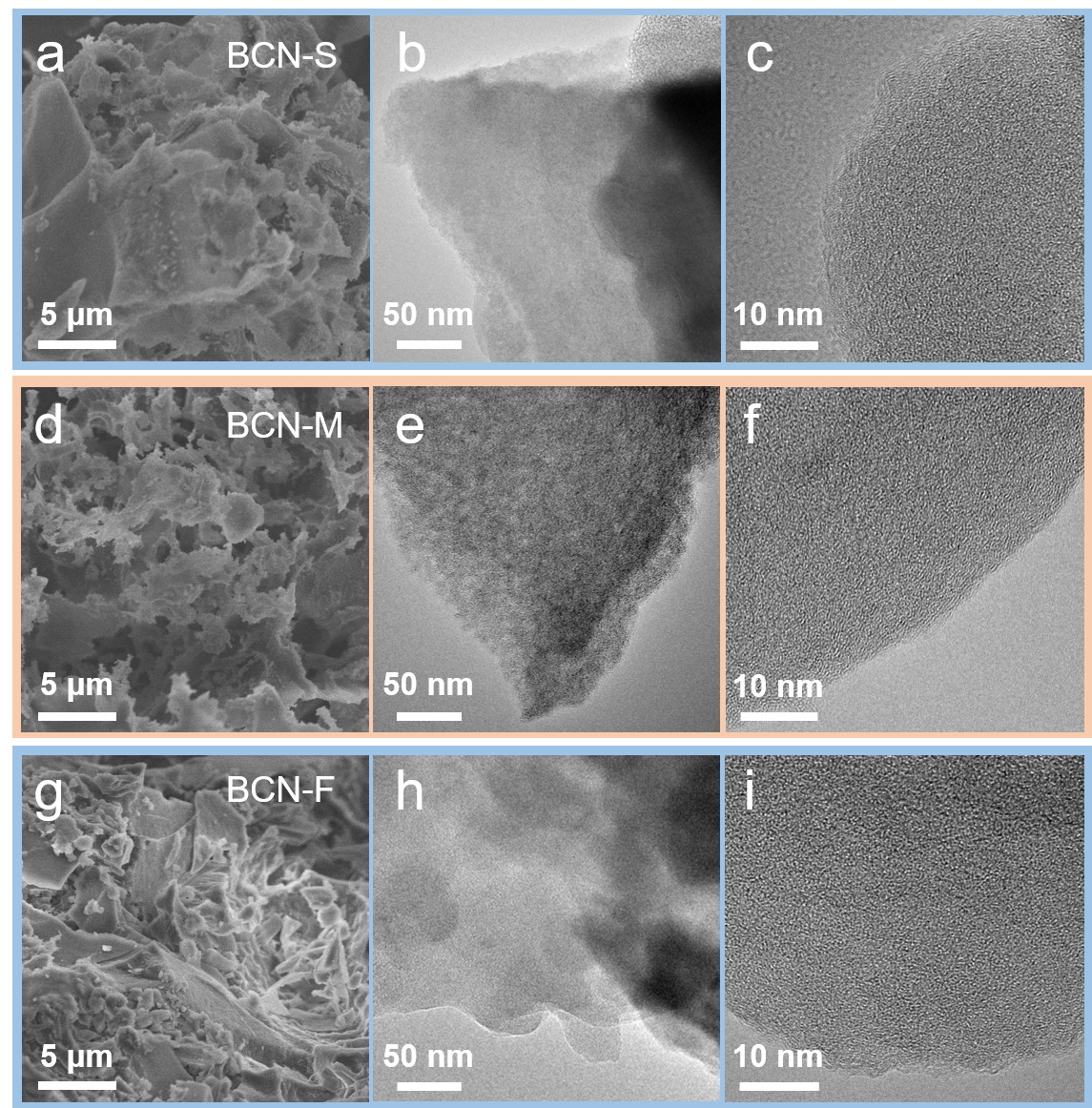


**Supplementary Figure 1.** Images of (**a**, **d**, **g**) SEM, (**b**, **e**, **h**) TEM and (**c**, **f**, **i**) HRTEM for BCN-S, BCN-M and BCN-F photocatalysts, respectively..

*
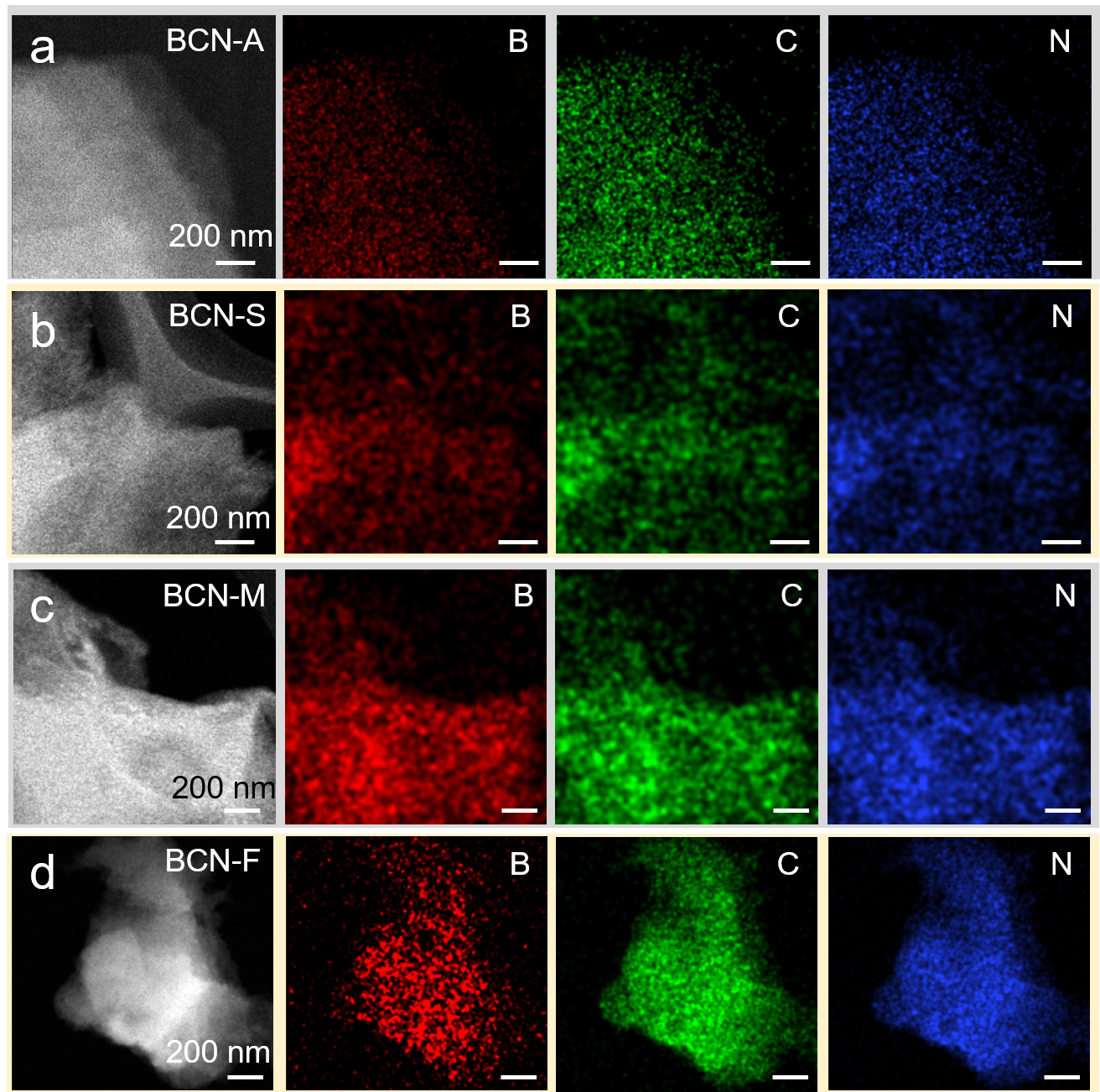
*

**Supplementary Figure 2.** EDS maps of (**a**) BCN-A, (**b**) BCN-S, (**c**) BCN-M, and (**d**) BCN-F photocatalysts.


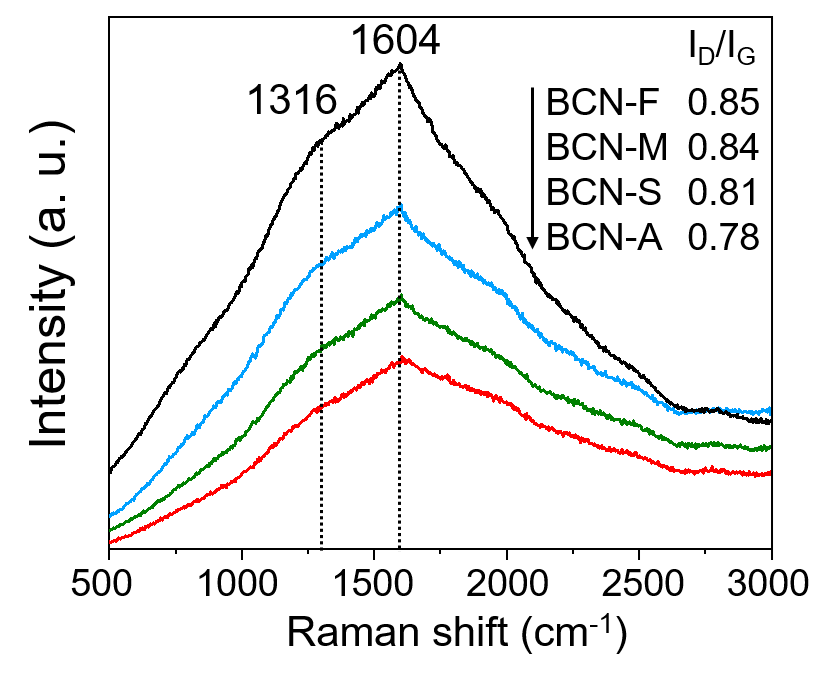


**Supplementary Figure 3.** Raman spectra of BCN photocatalysts.


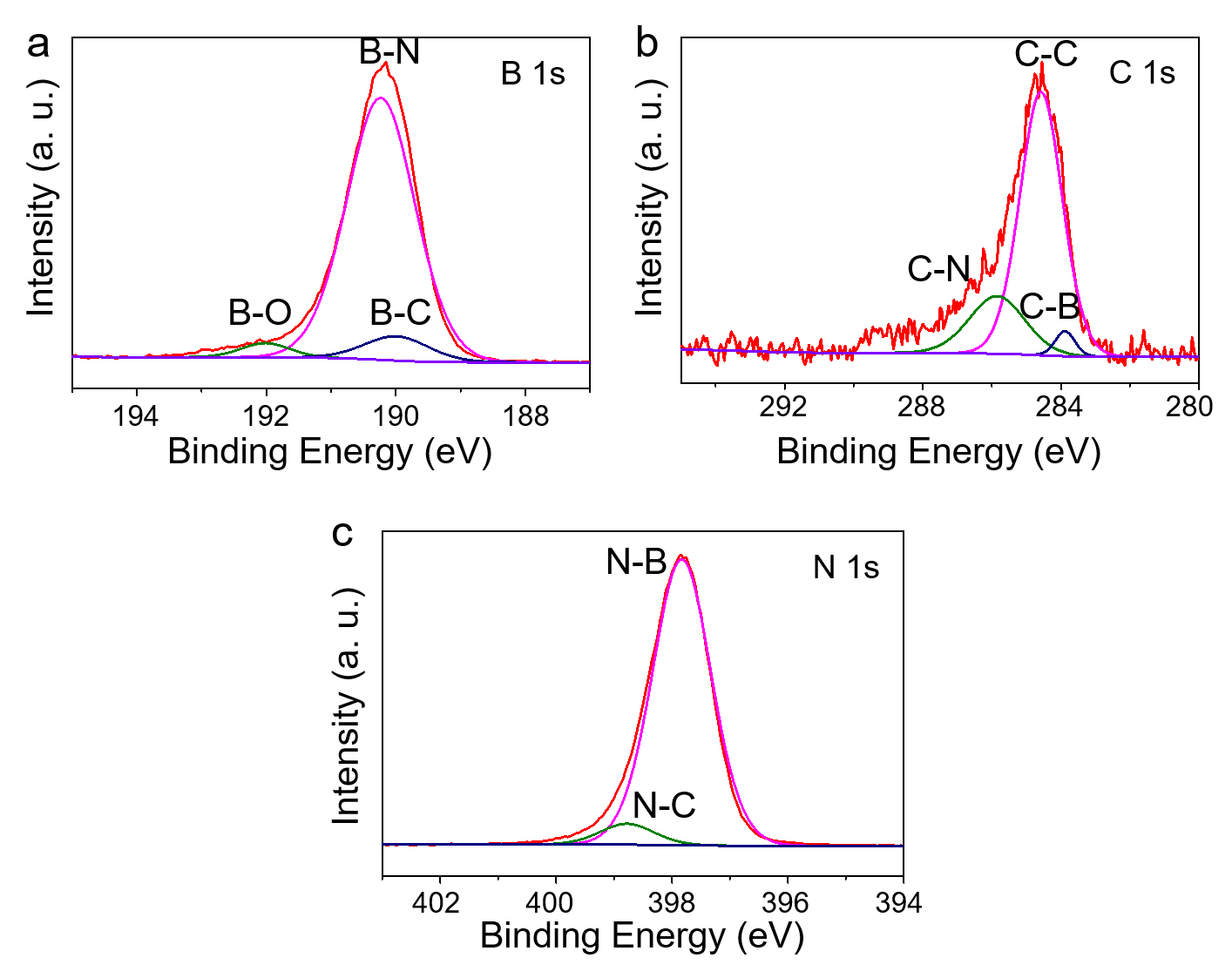


**Supplementary Figure 4.** High-resolution XPS spectra of (**a**) B 1s, (**b**) C 1s, and (**c**) N1s of BCN-A photocatalysts.


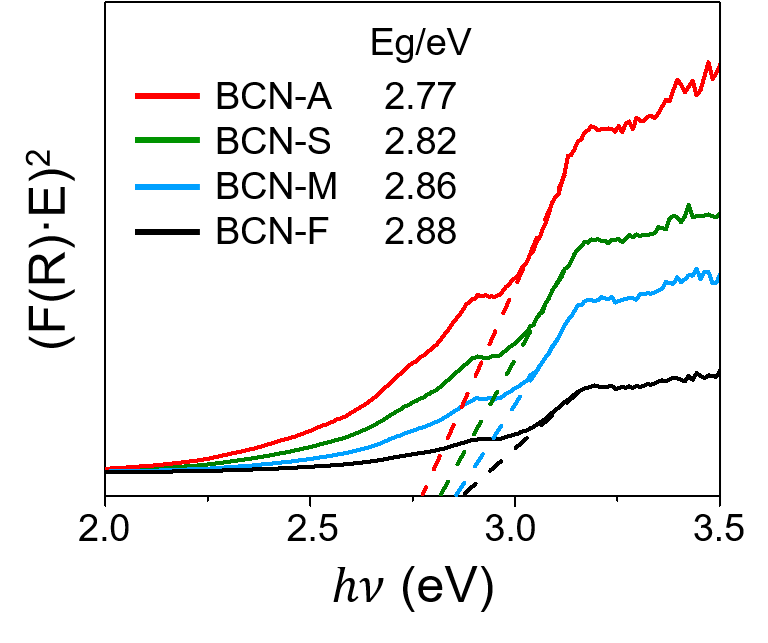


**Supplementary Figure 5.** Kubelka-Munk transformed reflectance spectra of BCN-A photocatalysts.

*
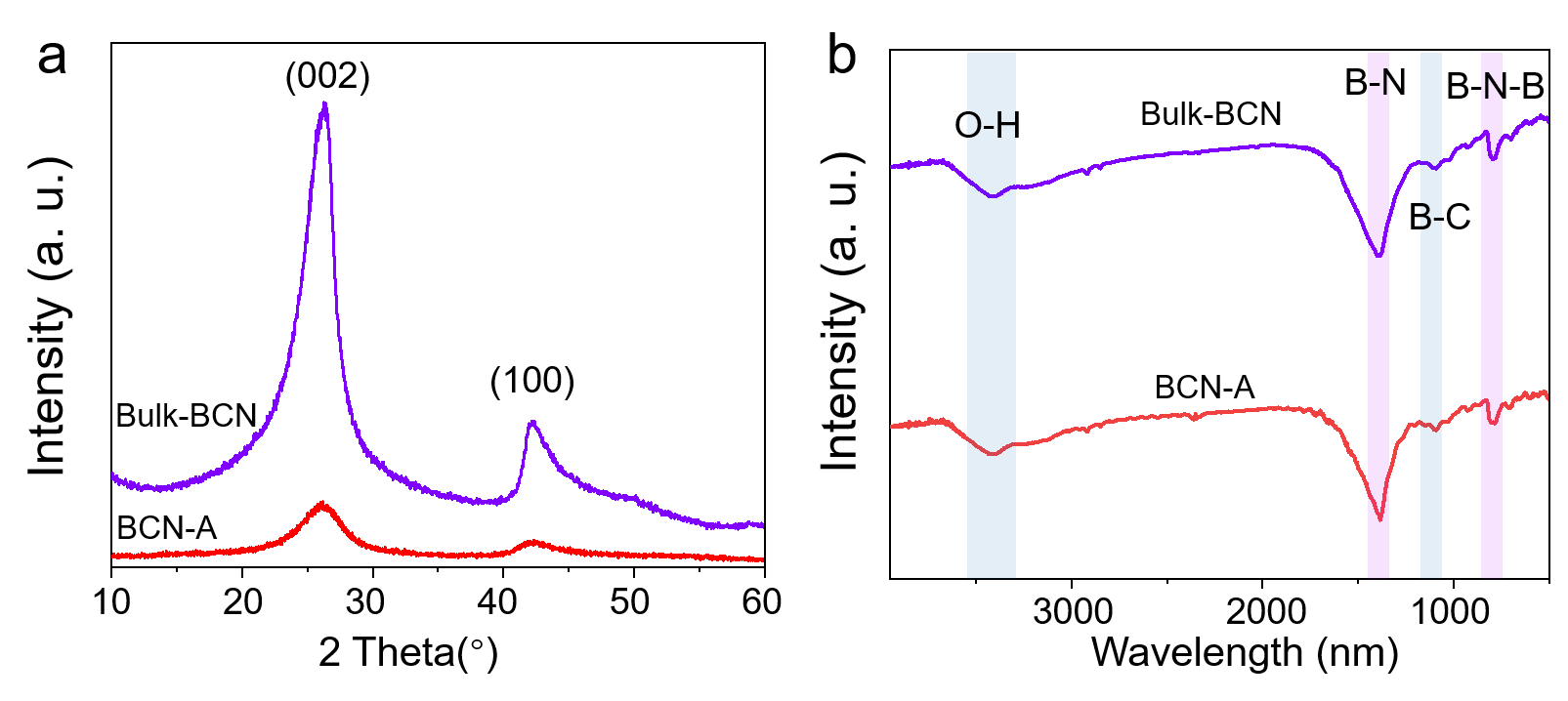
*

**Supplementary Figure 6.** (**a**) The XRD and (**b**) FT-IR spectra of BCN-A and bulk BCN photocatalysts.

The compositional and structural characteristics of both BCN-A and bulk BCN photocatalysts show remarkable similarities. An examination of the XRD spectra reveals that the bulk BCN also exhibits two prominent peaks located at 26.1° and 42.5° respectively, which are attributed to the (002) and (100) crystal planes of the graphitic-like crystal structure of h-BN. In addition, FT-IR spectroscopy confirms the disordered graphitic structure of the bulk BCN. This is evidenced by bands at 1380 and 798 cm^-1^, which are indicative of sp^2^-hybridized boron-nitrogen (B–N) in-plane transverse stretching vibrations and out-of-plane B–N–B bending vibrations, respectively. A minor peak observed at approximately 1093 cm^-1^ is indicative of B-C vibrational modes, implying the incorporation of carbon atoms into the boron nitride (BN) lattice. Additionally, a broad absorption feature between 3200-3600 cm^-1^ for the bulk BCN photocatalysts can be attributed to physically adsorbed H_2_O molecules. Furthermore, an evaluation of the hydrogen evolution reaction (HER) was carried out for the bulk BCN photocatalysts. The results shows a relatively low performance of hydrogen evolution rate with 12 μmol·h^-1^·g^-1^ in Fig. 4a and Supplementary Table 3.

*
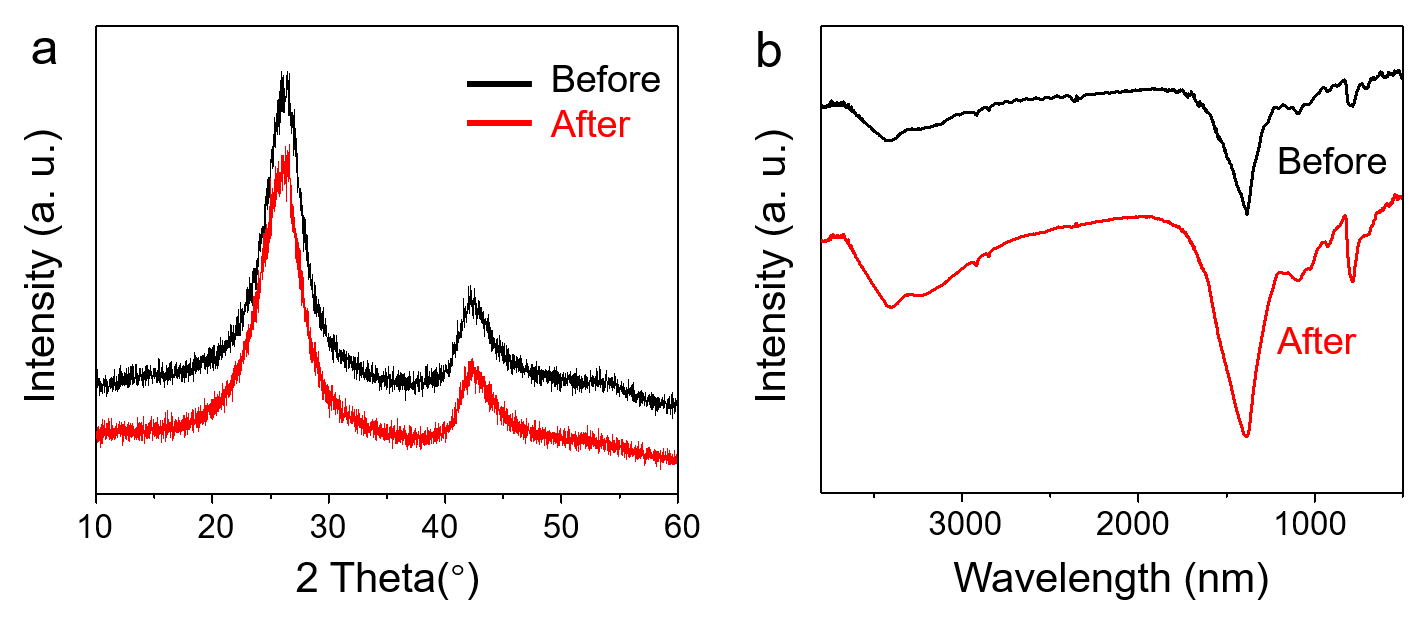
*

**Supplementary Figure 7.** (**a**) The XRD and (**b**) FT-IR spectra of BCN-A photocatalysts before and after the long-term stability test.

*
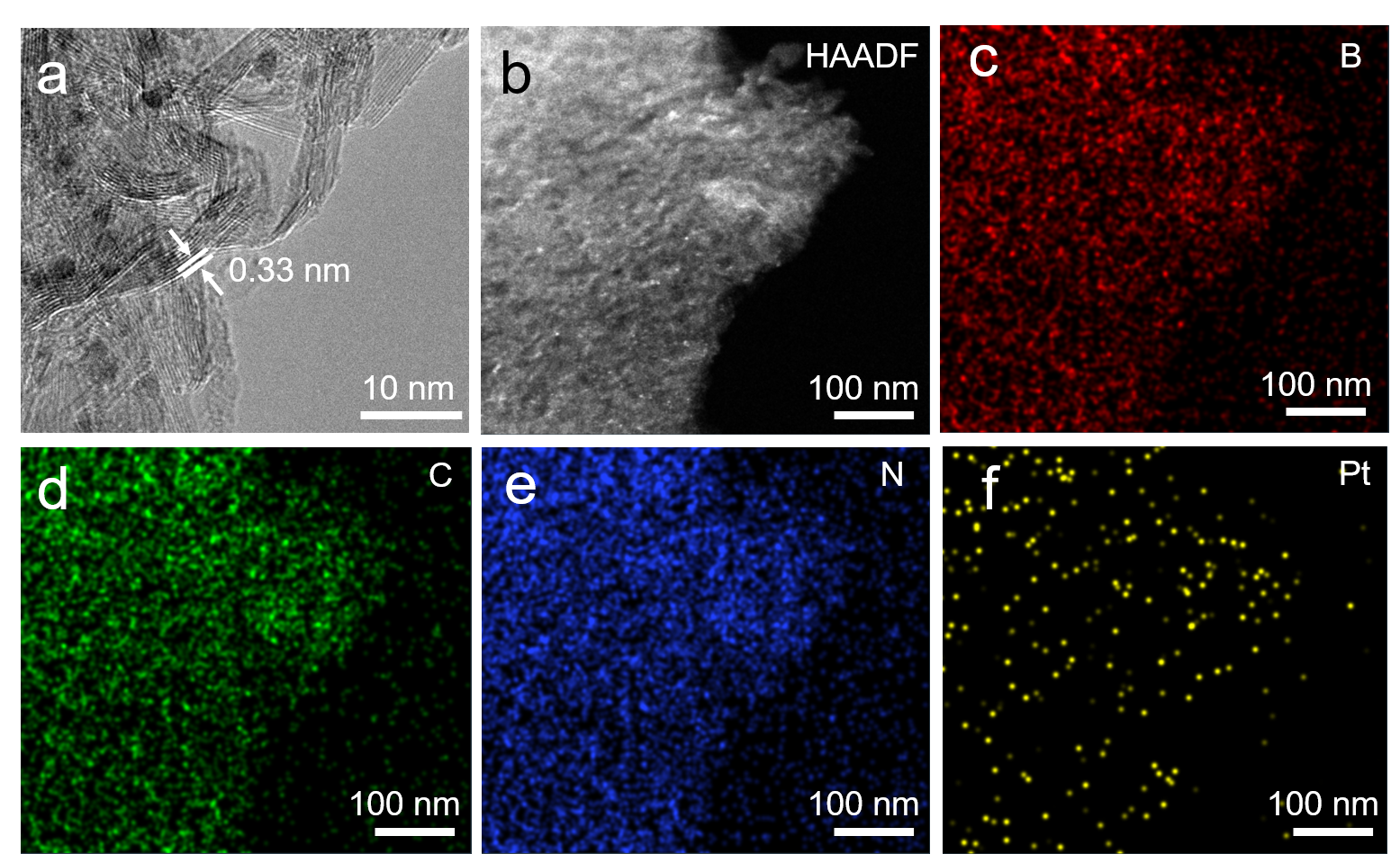
*

**Supplementary Figure 8.** (**a**) TEM and (**b-f**) EDS maps of BCN-A photocatalysts after the long-term stability test.

*
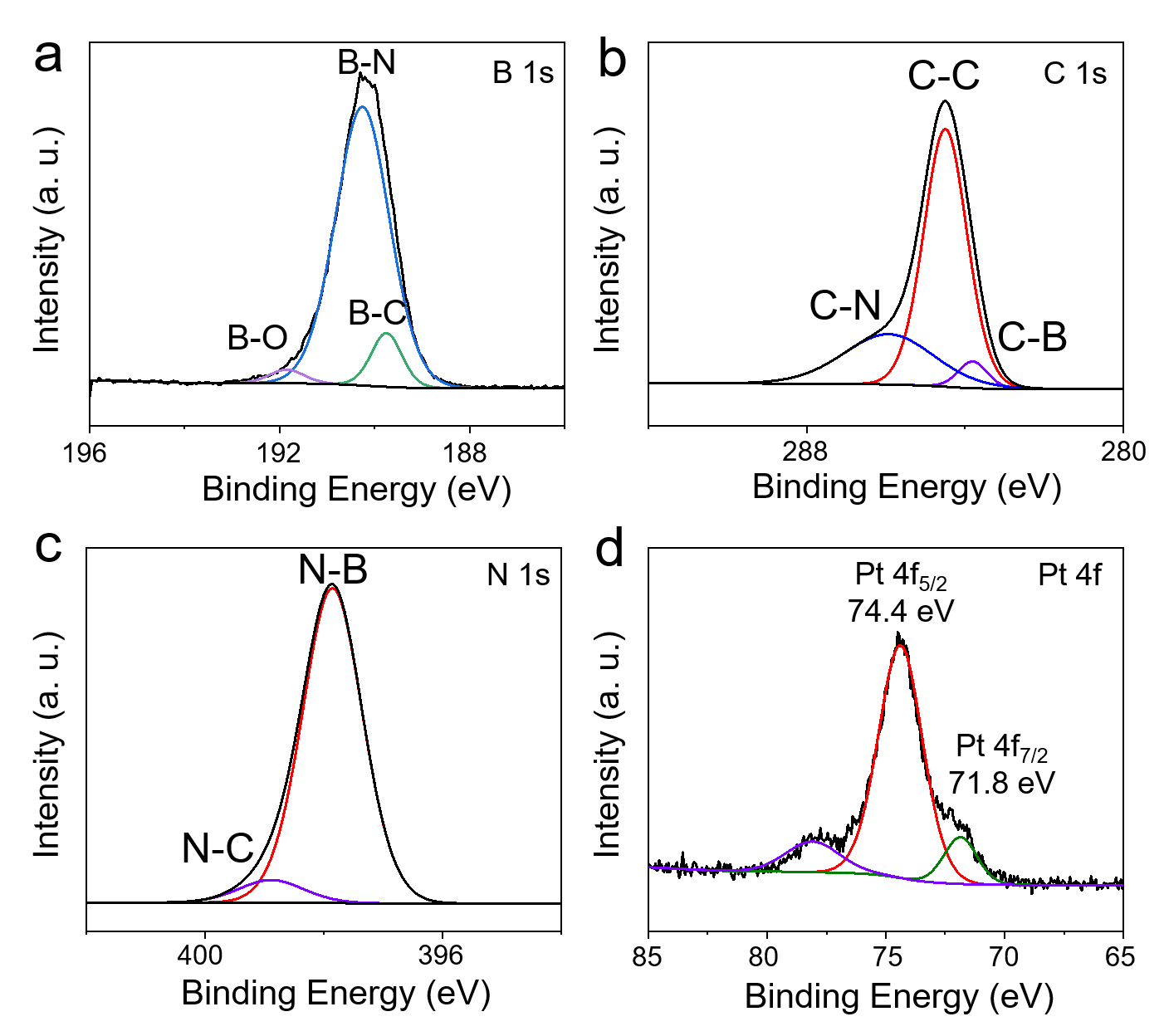
*

**Supplementary Figure 9.** High-resolution XPS spectra of (**a**) B 1s, (**b**) C 1s, (**c**) N1s , and (**d**) Pt 4f of BCN-A photocatalysts after the long-term stability test.

Although the characteristic signal peaks of Pt particles were not discerned in the XRD spectrum, this may be attributed to the substantive amount of Pt loading, which was beneath the detection threshold (Supplementary Fig. 7). However, subsequent to the reaction, the presence of Pt particles was ascertained through both TEM and XPS analyses. As illustrated in Supplementary Fig. 8, the reacted Pt particles were observed to be adhered to the surface of BCN-A photocatalysts, and a lattice fringe of 0.33 nm persisted for BCN-A photocatalysts, signifying the commendable stability of BCN-A photocatalysts. Follow-up XPS assessments further corroborated the presence of Pt, with no conspicuous shifts evident in the XPS peak positions of B, C, and N, nor were there any additional impurity peaks detected (Supplementary Fig. 9), further attesting to the excellent stability of BCN-A. No post-reaction triethanolamine was discerned in the FT-IR spectra, potentially due to the depletion of triethanolamine during the collection process of the post-reaction samples, which involved repeated washing and drying, consequently resulting in no measurable FT-IR peak values for triethanolamine in the post-reaction specimens.

*
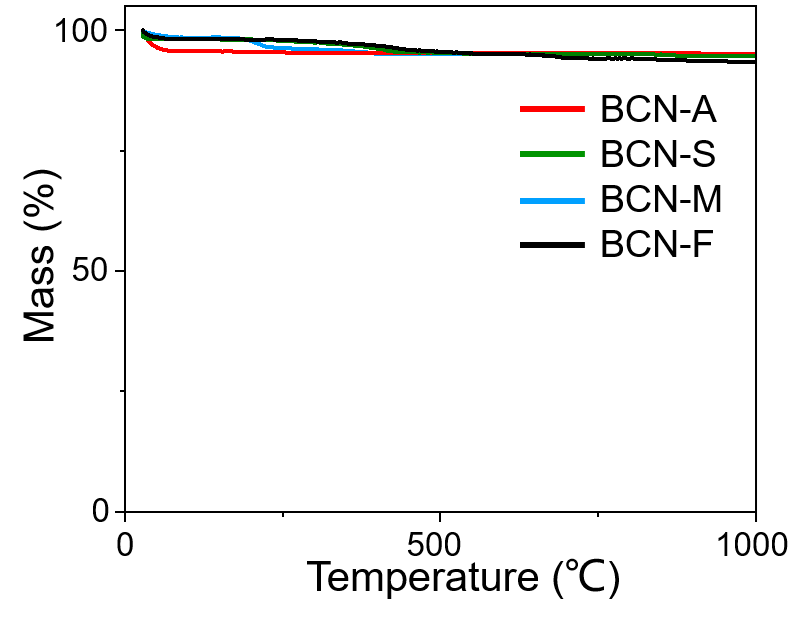
*

**Supplementary Figure 10.** The image of TGA analysis for BCN photocatalysts.

**Supplementary Tables**

**Supplementary Table 1.** Elemental analysis results from EDS data for BCN photocatalysts.

| **Sample** | **Boron (at%)** | **Carbon (at%)** | **Nitrogen (at%)** | **Oxygen (at%)** | **Chemical formula**  **(the oxygen element is omitted)** |
| --- | --- | --- | --- | --- | --- |
| BCN-A | 42.2 | 30.6 | 21.5 | 5.7 | B_1.38_CN_0.70_ |
| BCN-S | 41.3 | 31.2 | 20.7 | 6.8 | B_1.32_CN_0.66_ |
| BCN-M | 40.2 | 32.6 | 19.8 | 7.4 | B_1.23_CN_0.61_ |
| BCN-F | 39.4 | 33.8 | 18.6 | 8.2 | B_1.17_CN_0.55_ |

**Supplementary Table 2.** The data of specific surface area for BCN photocatalysts.

| **Sample** | **Specific surface area /m^2^g^-1^** |
| --- | --- |
| BCN-A | 205 |
| BCN-S | 35 |
| BCN-M | 34 |
| BCN-F | 28 |

**Supplementary Table 3.** Summary of BCN photocatalysts for hydrogen evolution rate (HER) in the literatures.

| **Sample** | **Reation conditions** | **Light source** | **HER**  **(μmolh^-1^g^-1^)** | **Ref.** |
| --- | --- | --- | --- | --- |
| BCN-A nanosheets | 300 W Xe lamp,  λ > 420 nm | 1.0 wt% Pt,  aqueous TEOA  solution (10 vol%) | 110 | This work |
| Bulk BCN |  |  | 12 |  |
| BCN-S |  |  | 46 |  |
| BCN-M |  |  | 34 |  |
| BCN-F |  |  | 22 |  |
| BCN nanosheets | 300 W Xe lamp,  λ > 420 nm | 1.0 wt% Pt,  aqueous TEOA  solution (10 vol%) | 82 | Ref^1^ |
| BCN tubes | 300 W Xe lamp,  λ > 420 nm | 1.0 wt% Pt,  aqueous TEOA  solution (10 vol%) | 56 | Ref^2^ |
| BCN nanosheets | 300 W Xe lamp,  λ > 420 nm | 1.0 wt% Pt,  aqueous TEOA  solution (10 vol%) | 76 | Ref^3^ |
| BCN-TiO_2_  nanocomposites | 300 W Xe lamp,  λ > 420 nm | Pt,  aqueous TEOA  solution (20 vol%) | 19.7 | Ref^4^ |

**Supplementary References**

1. Chen, L. *et al.* Template-free synthesis of carbon-doped boron nitride nanosheets for enhanced photocatalytic hydrogen evolution. *Appl. Catal. B- Environ.* **241**, 246-255 (2019).

2. Chen, L. & Wang, X. Bio-templated fabrication of metal-free boron carbonitride tubes for visible light photocatalysis. *Chem. Commun.* **53**, 11988-11991 (2017).

3. Zhang, M. *et al.* Molten salt assisted assembly growth of atomically thin boron carbon nitride nanosheets for photocatalytic H_2_ evolution. *Chem. Commun.* **56**, 2558-2561 (2020).

4. Xing, X. *et al.* Z-scheme BCN-TiO_2_ nanocomposites with oxygen vacancy for high efficiency visible light driven hydrogen production. *Int. J. Hydrogen Energ* **42**, 28434-28444 (2017).
